# Clinical relevance of the 3-cm threshold in sigmoid diverticulitis with abscess: consensus or quandary?

**DOI:** 10.1007/s00384-024-04682-z

**Published:** 2024-07-12

**Authors:** Sascha Vaghiri, Stephan Oliver David, Ahmad Baktash Sultani, Sami Alexander Safi, Wolfram Trudo Knoefel, Dimitrios Prassas

**Affiliations:** 1 Department of Surgery (A), Heinrich-Heine-University, Medical Faculty and University Hospital Duesseldorf, Duesseldorf, Germany; 2 Department of Surgery, Katholisches Klinikum Essen, Philippusstift, Teaching Hospital of Duisburg-Essen University, Huelsmannstrasse 17, 45355 Essen, Germany

**Keywords:** Diverticular abscess, Abscess size, Operative outcome, Therapy failure

## Abstract

**Purpose:**

Diverticular abscess is a common manifestation of acute complicated diverticulitis. We aimed to analyze the clinical course of patients with diverticular abscess initially treated conservatively.

**Methods:**

All patients with diverticular abscess undergoing elective or urgent/emergency surgery from October 2004 to October 2022 were identified from our institutional database. Depending on the abscess size, patients were divided into group A (≤ 3 cm) and group B (> 3 cm). Conservative treatment failure was defined as clinical deterioration, persistent or recurrent abscess, or urgent/emergency surgery. Baseline characteristics and short-term perioperative outcomes were recorded and compared between both groups. Uni- and multivariate analyses were conducted to identify determinants of conservative treatment failure and overall ostomy formation.

**Results:**

A total of 105 patients were enrolled into group A (*n* = 73) and group B (*n* = 32). Uni- and multivariate analyses revealed abscess size as the only significant factor of conservative therapy failure [OR 9.904; *p* < 0.0001], while overall ostomy formation was significantly affected by an increased body mass index (BMI) [OR 1.366; *p* = 0.026]. There were no significant differences in perioperative outcome with the exception of a longer total hospital stay in patients managed with abscess drainage compared to antibiotics alone prior surgery in group B (*p* = 0.045).

**Conclusion:**

Abscess diameter > 3 cm is not just an arbitrary chosen cut-off value for drainage placement but has a prognostic impact on medical treatment failure in patients with complicated acute diverticulitis. In this subgroup, the choice between primary drainage and antibiotics does not appear to influence outcome at the cost of prolonged hospital stay after drainage insertion.

**Supplementary Information:**

The online version contains supplementary material available at 10.1007/s00384-024-04682-z.

## Introduction

Diverticular abscess is the most common manifestation of complicated diverticular disease which occurs in 15–40% of patients with acute sigmoid diverticulitis [1, 2]. The 30-day mortality rate associated with diverticular abscess is 8.7%, according to large Danish register-based cohort study with 3148 patients [3]. Depending on abscess size and location, medical treatment with antibiotic administration and/or percutaneous interventional abscess drainage represents a well-proven non-operative strategy in patients with diverticular abscess formation [2]. The failure rate of initial conservative management ranges between 13.9 and 20% [4–6]. Abscess size is recognized as a predictive outcome factor following initial medical therapy [7]. In a large multicenter Dutch study [8], abscess diameters of ≥ 3 or 5 cm were significantly associated with higher short-term treatment failure and emergency surgery, respectively. Other studies [9, 10] revealed higher recurrence rates after medical therapy of abscesses larger than 5 cm. Currently, there is no uniform consensus on the most appropriate abscess size threshold for interventional drainage placement as the majority of results come from heterogeneous studies with low level of evidence [11]. The latest German [12, 13] and ASCRS [2] guidelines recommend drainage of larger abscesses (> 3 cm) if technically feasible because sole antibiotic therapy is associated with a higher failure rate up to 34% [14, 15]. In contrast, a study from Finland demonstrated similar overall failure rates of antibiotics versus percutaneous abscess drainage in ≥ 4 cm abscess size [16]. Of note, in a meta-analysis with 42 included studies, the rate of recurrence in patients with abscess drainage was lower than antibiotics alone (15.9% versus 22.2%) [4]. Most of these recurrences, especially after abscess drainage, were again complicated relapses (71.1%), requiring subsequent urgent surgery in 29.2% [9]. Mortality rates after emergent resection during recurrence were 4.6% as compared to only 0.3% following resection in a delayed elective setting based on a large American database query [17].

Remarkably, there is restricted data in the current literature analyzing the effects of an abscess size equal or larger than 3 cm, initially treated conservatively, on surgical outcome [14]. This data demonstrated no significant differences in the rate of elective colectomy, overall morbidity and stoma creation, and length of hospital stay between antibiotic diverticular abscess coverage and percutaneous drainage in a cohort of 146 patients although median abscess size was significantly larger in the drainage group.

Hence, the primary objective of this study was to assess the course of patients with CT-verified diverticular abscess undergoing initial conservative treatment followed by either elective or emergency resection and to determine predictive factors of medical therapy failure and overall ostomy formation at the time of surgery with special emphasis on the cut-off abscess size of 3 cm.

## Material and methods

### Patient collective and study design

In this single-center retrospective cohort study, all patients with complicated acute sigmoid diverticulitis and abscess formation treated from October 2004 to October 2022 at the Department of General, Visceral and Pediatric Surgery at the Medical Faculty and University Hospital Duesseldorf, Germany, were identified from a large prospectively maintained database. Exclusion criteria were age < 18 years, known or incidental finding of colorectal cancer, free perforation, covert perforation with extraluminal air bubbles but without abscess detection, primary surgery after admission, and medical management without subsequent elective or emergency sigmoidectomy. In all patients with suspected sigmoid diverticulitis upon initial presentation, the diagnosis of diverticular abscess was documented via computed-tomography (CT) imaging. After interdisciplinary discussion with an interventional radiology specialist, percutaneous CT-guided drainage placement was conducted depending on abscess diameter and location and the overall condition of the patient. Our standardized antibiotic regimen included daily intravenous (i.v.) administration of ceftriaxone/metronidazole or ciprofloxacin/metronidazole or piperacillin/tazobactam in case of clinical deterioration for at least 5–7 days. All patients were initially put on nil per os and subsequently started stepwise oral diet consumption once they tolerated solid food and signs of disease improvement were apparent. Prolonged episodes of fasting were bridged with total parenteral nutrition. The abscess drain was flushed 2–3 times daily with saline until the secretion became serosanguinous. After resolution of symptoms and normalization of inflammatory parameters, the drainage was removed if daily output ceased < 50 ml over 24 h. Prior drainage removal, all patients underwent routine CT or ultrasound follow-up imaging to rule out abscess remnant. Failure of initial conservative treatment (either antibiotics or drainage/antibiotics) was defined as clinical deterioration with abdominal complaints and tenderness, constantly elevated or raising inflammation markers (white blood count, C-reactive protein), persistent or recurrent diverticular abscess, and diverticulitis associated re-admission within 30 days since index admission prompting emergency or urgent sigmoid resection. Patients with successful medical treatment either underwent elective sigmoidectomy at the same hospital stay or were discharged with oral antibiotics and regular follow-up clinic appointments and were consecutively offered surgery in the inflammation-free interval after 4–6 weeks since the last flair based on current guideline recommendations [2, 12, 13].

This article was written in strict accordance with the latest version of the Declaration of Helsinki and the “Strengthening the Reporting of Observational Studies in Epidemiology” (STROBE) checklist for observational studies [18]. Informed consent was waived because no data regarding the cases were disclosed. Approval of the local ethics committee of the Medical Faculty, Heinrich-Heine-University Duesseldorf, Germany (study no. 2021–1346), was granted prior study initiation.

### Data collection and group definition

After reviewing medical and operative charts of each included patient, the following parameters and information were collected: (1) demographic data (i.e., age, gender, body mass index (BMI), and American Society of Anesthesiologists (ASA) classification) and comorbidities (e.g., cardiovascular and metabolic disease or immunosuppression), number of previous attacks, radiological assessment of the ongoing complicated diverticulitis attack, time interval from acute onset to surgery, and laboratory parameters (including C-reactive protein (CRP), white blood count (WBC), hemoglobin, and thrombocytes); (2) detailed conservative treatment strategy (antibiotics versus drainage), failure rates of initial medical treatment as defined previously, surgical approach, and intraoperative course (e.g., conversion rate or stoma creation, duration of surgery); (3) postoperative surgical complications (e.g., wound infection, anastomotic leak or stenosis, postoperative ileus, incisional hernia, ureter lesion, and intra-abdominal abscess formation) or medical complications (e.g., sepsis, pneumonia, renal failure, or cardiovascular events), and re-operation or intervention; and (4) in-hospital mortality, (total and postoperative) length of hospital stay, and ostomy reversal rates. Major morbidity was classified as Clavien-Dindo ≥ 3a [19]. All eligible patients for analysis were divided into two separate groups according to the defined abscess size cut-off diameter of 3 cm: group A with micro-abscess (≤ 3 cm) versus group B with macro-abscess (> 3 cm).

### Statistical analysis and outcomes

The primary outcome of interest was failure of conservative treatment and urgent/emergency surgery, respectively. The secondary outcome was overall ostomy creation after elective or urgent/emergency surgery. Statistical analysis was performed using SPSS 25.0 software program (Statistical Package for Social Sciences; SPSS Inc., Chicago, IL, USA). Descriptive statistics for continuous variables were presented as mean ± standard deviation (SD) and compared using the Mann–Whitney *U* or Student *t*-test. Categorical data were summarized as frequencies (%). Comparison of categorical variables was conducted by applying the Pearson *χ*^2^ test or Fisher’s test, as appropriate. Risk factors for failure of conservative therapy and overall ostomy formation were identified using univariate analysis. Variables with *p* value less than 0.05 were entered in the multivariable logistic regression model (Enter method). Hazard ratios (HRs) with 95% confidence intervals (CIs) were estimated. A *p* value < 0.05 was considered to be significant.

## Results

### Patient and disease characteristics

**Fig. 1:**
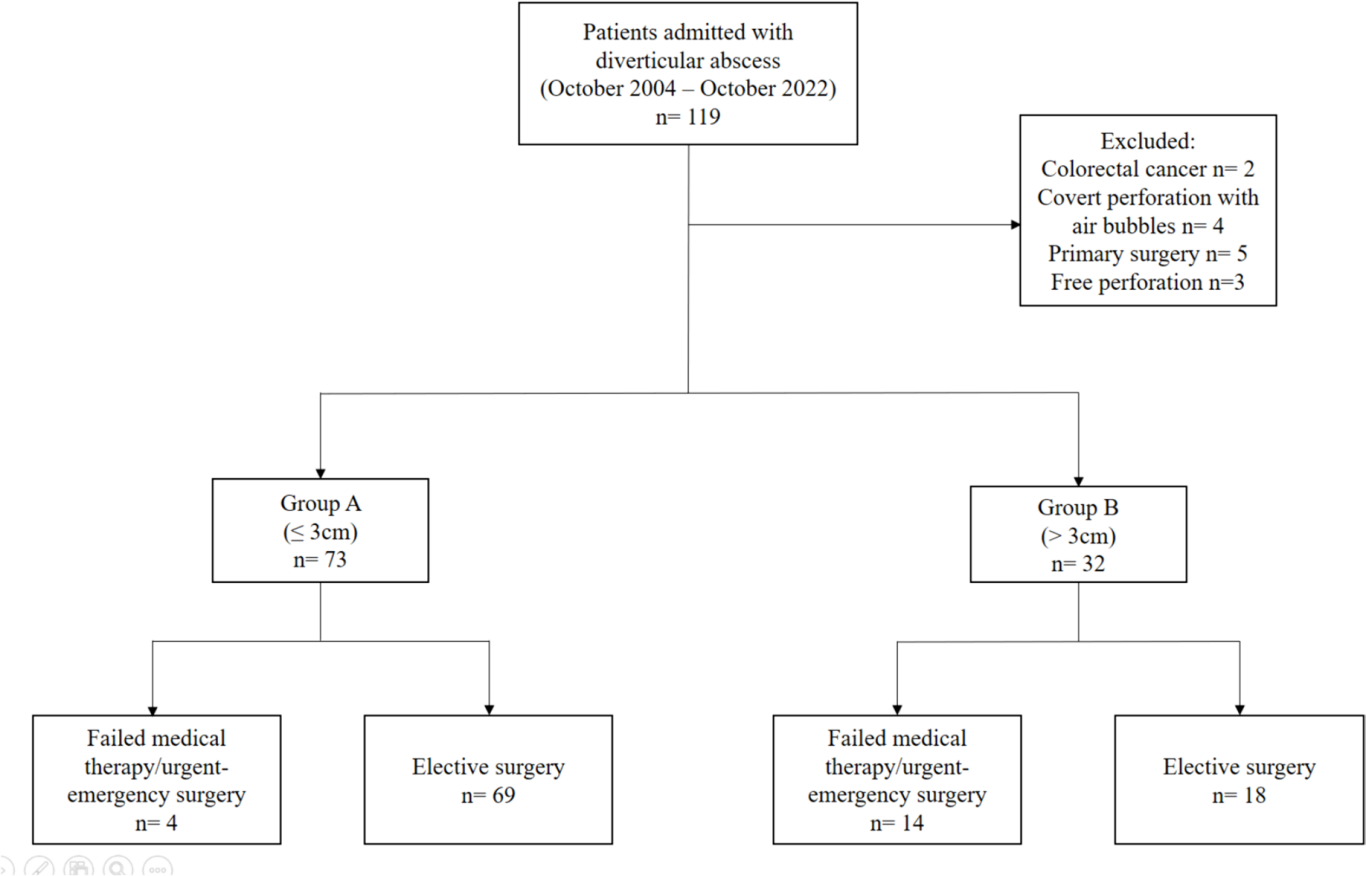
Flowchart diagram of patient selection and analysis

Between October 2004 and October 2022, a total of 119 patients with diverticular abscess were admitted to our surgical department. After exclusion of 14 patients which did not meet the eligibility criteria, the remaining 105 were included in the final analysis as illustrated in Fig. 1. Furthermore, of this population, 73 patients with diverticular abscess size ≤ 3 cm were defined as group A, while 32 patients suffering from a complicated acute diverticulitis attack with an abscess diameter > 3 cm on CT imaging were summarized in group B. The preoperative patient demographics and the disease course are demonstrated in Table 1. There were significantly more female patients in group B compared to group A [*n* = 21 (65.63%) versus *n* = 28 (38.36%); *p* = 0.012]. However, other characteristics such as age, BMI, and overall health condition (reflected by the ASA score) were not significantly different between both groups. Interestingly, among the relevant comorbidities, arterial hypertension and chronic kidney disease were significantly more prevalent in the macro-abscess group (62.56% versus 35.62% (*p* = 0.018), respectively, 18.75% versus 4.11% (*p* = 0.022)). At the time of admission, the mean value of the inflammatory parameters CRP and WBC was significantly higher in group B in comparison to group A [CRP (mg/dl): group B 9.050 ± 8.293 versus group A 5.187 ± 7.328 (*p* = 0.019), respectively, WBC (× 1000/µl): group B 12.356 ± 5.488 versus group A 9.970 ± 4.443 (*p* = 0.021)]. Group A patients had a mean abscess diameter of 1.172 ± 0.772 cm, which was significantly smaller than the mean observed abscess size of 5.471 ± 2.024 cm in group B (*p* < 0.0001). While the majority of abscesses in group A were located in the paracolic region (93.15%), pelvic, retroperitoneal, and distant abscess formations were significantly higher recognized in group B patients (68.75%) (*p* < 0.0001). Concomitant abscess complications including fistula (6.25%) and stenosis (3.13%) were only observed in group B (*p* = 0.026). More than half of the patients with a macro-abscess (56.25%) underwent interventional CT-guided abscess drainage insertion compared to only one patient (1.37%) in group A (*p* < 0.0001). Considering the primary study outcome, the occurrence of conservative therapy failure leading to subsequent urgent/emergency surgery was significantly higher in group B in comparison to group A (43.75% versus 5.48%, *p* < 0.0001). True abscess recurrences within 30 days since admission were noticed in one group A (1.37%) and four group B (12.50%) patients (*p* = 0.029). If the macro-abscess group is observed separately, the treatment failure rate of the patients with drainage was not significantly different from those patients who were managed with antibiotics alone (drainage 55.56% versus antibiotics 28.57%, *p* = 0.165) (Table 1 suppl.). Elective surgery after successful medical management by means of complete symptom and abscess resolution was performed early electively (within 6 weeks) in 69.85% of group A and 46.88% of group B patients, respectively, while a late elective procedure (> 6 weeks) was predominantly performed in group A with 24.66% as opposed to 9.38% in group B (*p* = 0.543).

**Table 1:** Preoperative patient demographics/data and disease characteristics

	Group A *n* = 73	Group B *n* = 32	*p* value
Variables			
Gender (male/female) (*n*; %)	45/28 (61.64/38.36)	11/21 (34.38/65.63)	**0.012**
Age (years) (mean ± SD)	55.671 ± 12.129	55.218 ± 12.605	0.862
BMI (kg/m^2^) (mean ± SD)	27.313 ± 5.417	28.665 ± 8.674	0.420
ASA score (*n*; %)			0.644
I II III IV	16 (21.92) 37 (50.68) 18 (24.66) 2 (2.74)	5 (15.63) 16 (50.0) 11 (34.38) 0 (0)	
Comorbidities (*n*; %)			
Diabetes mellitus	5 (6.85)	2 (6.25)	1.000
Arterial hypertension	26 (35.62)	20 (62.50)	**0.018**
Chronic kidney disease	3 (4.11)	6 (18.75)	**0.022**
Immunosuppression	8 (10.96)	7 (21.88)	0.223
Laboratory values			
CRP (mg/dl) (mean ± SD)	5.187 ± 7.328	9.050 ± 8.293	**0.019**
WBC (× 1000/µl) (mean ± SD)	9.970 ± 4.443	12.356 ± 5.488	**0.021**
Hemoglobin (g/dl) (mean ± SD)	13.577 ± 2.157	12.759 ± 1.864	0.067
Thrombocytes (× 1000/µl) (mean ± SD)	279.098 ± 152.855	339.687 ± 116.794	**0.049**
Number of flairs (mean ± SD)	1.753 ± 0.968	1.656 ± 0.901	0.630
Abscess size (cm) (mean ± SD)	1.172 ± 0.772	5.471 ± 2.024	** < 0.0001**
Abscess localization (*n*; %)			** < 0.0001**
Paracolic Pelvic Retroperitoneal Distant	68 (93.15) 5 (6.85) 0 (0) 0 (0)	10 (31.25) 15 (46.88) 4 (12.50) 3 (9.38)	
Abscess complication (*n*; %)			**0.026**
Fistula Stenosis	0 (0) 0 (0)	2 (6.25) 1 (3.13)	
Interventional drainage (*n*; %)	1 (1.37)	18 (56.25)	** < 0.0001**
Number of abscess drains (*n*; %)			1.000
1 2 > 2	1 (1.37) 0 0	14 (43.75) 3 (9.38) 1 (3.13)	
Failed conservative therapy/urgent-emergency surgery (*n*; %)	4 (5.48)	14 (43.75)	** < 0.0001**
Abscess recurrence (*n*; %) Persistent abscess (*n*; %) Lab/clinical deterioration (*n*; %)	1 (1.37) 1 (1.37) 2 (2.74)	4 (12.50) 5 (15.63) 5 (15.63)	** < 0.0001**
Time interval surgery to last attack—all patients (days) (mean ± SD)	25.534 ± 25.405	17.875 ± 20.359	0.105
Surgery at index admission (*n*; %)	39 (53.42)	22 (68.75)	0.197
Time interval urgent/emergency surgery to admission (days) (mean ± SD)	5.750 ± 7.041	8.428 ± 7.653	0.540
Timing elective surgery (*n*; %)			0.543
Early (< 6 weeks) Late (≥ 6 weeks)	51 (69.86) 18 (24.66)	15 (46.88) 3 (9.38)	

*ASA* American Society of Anesthesiologists, *BMI* body mass index, *CRP* C-reactive protein, *WBC* white blood count

### Intraoperative course and postoperative outcome

The intraoperative course respectively postoperative outcomes is highlighted in Table 2. Notably, a laparoscopic access was chosen more frequently as the preferred surgical approach in group A (78.08%) compared to group B (50.0%) (*p* = 0.006). The rate of conversion to an open procedure was significantly higher in group B patients undergoing primary laparoscopy (43.75% versus 17.54%, *p* = 0.044). In group A, sigmoid resection with a primary anastomosis was performed in 91.78% and in 5.48% protective diversion ostomy was additionally constructed. A Hartmann procedure was necessary in two patients (2.74%). In contrast, 18.75% of patients in group B underwent primary resection with anastomosis and protective ostomy. The rate of Hartmann resection was also higher in group B (18.75%). The mean operative duration was comparable between both groups (group A 281.671 ± 79.950 min versus group B 277.093 ± 77.521 min, *p* = 0.786). Analyzing the postoperative outcome, we found no significant differences in the frequency of overall and major morbidities in both groups but the rate of overall ostomy formation (primary or secondary due to complications) was significantly higher in patients with macro-abscesses (40.63% versus 12.33%, *p* = 0.002). Another difference was noticed in the duration of total and postoperative hospital stay which were both significantly longer in group B patients (*p* < 0.05). Comparison of postoperative outcomes of drainage versus antibiotics in group B revealed no statistically significant differences (Table 1 suppl.). However, total hospital stay was significantly prolonged in patients with abscess drainage as opposed to antibiotics alone (*p* = 0.045). Of note, we recorded zero in-hospital mortality during the designated study period.

**Table 2:** Intra- and postoperative course

	Group A *n* = 73	Group B *n* = 32	*p* value
**Variables**			
Route of access (*n*; %)			**0.006**
Open Laparoscopic	16 (21.92) 57 (78.08)	16 (50.0) 16 (50.0)	
Conversion to open surgery (*n*; %)	10/57 (17.54)	7/16 (43.75)	**0.044**
Surgical procedure (*n*; %)			**0.002**
Hartmann resection Anastomosis without ostomy Anastomosis with protective ostomy	2 (2.74) 67 (91.78) 4 (5.48)	6 (18.75) 20 (62.50) 6 (18.75)	
Type of ostomy (*n*; %)			1.000
Ileostomy Colostomy	2 (2.74) 2 (2.74)	3 (9.38) 3 (9.38)	
Operative time (min) (mean ± SD)	281.671 ± 79.950	277.093 ± 77.521	0.786
Secondary ostomy (*n*; %)	3 (4.11)	1 (3.13)	1.000
Overall ostomy formation (*n*; %)	9 (12.33)	13 (40.63)	**0.002**
Overall postop. morbidity (*n*; %)	34 (46.58)	17 (53.13)	0.672
Major morbidity (CD ≥ 3a) (*n*; %)	12 (16.44)	9 (28.13)	0.191
Wound infection (*n*; %)	25 (34.25)	15 (46.88)	0.276
Anastomotic leak (*n*; %)	4/71 (5.63)	1/26 (3.85)	1.000
Re-operation (*n*; %)	7 (9.59)	5 (15.63)	0.506
Postoperative ileus (*n*; %)	5 (6.85)	4 (12.50)	0.450
Intra-abdominal abscess (*n*; %)	0 (0)	2 (6.25)	0.091
Trocar/incisional hernia (*n*; %)	1 (1.37)	1 (3.13)	0.519
Ureter lesion (*n*; %)	1 (1.37)	1 (3.13)	0.519
Postop. LOS (days) (mean ± SD)	13.068 ± 9.176	18.218 ± 16.840	**0.046**
Total LOS (days) (mean ± SD)	18.506 ± 11.030	27.531 ± 18.185	**0.013**
Ostomy reversal (*n*; %)	7/9 (77.78)	9/13 (69.23)	1.000

*CD* Clavien-Dindo, *LOS* length of hospital stay

### Uni- and multivariate analyses of predictive factors for treatment failure and overall ostomy formation

Table 3 presents the uni- and multivariate analyses of predictive variables for conservative treatment failure. Accordingly, hypertension, chronic kidney disease, and abscess size were associated to medical treatment failure with a *p* value < 0.05. After multivariate analysis of the above-mentioned variables, only abscess size (≤ 3 cm versus > 3 cm) [OR 9.904, 95% CI (2.778–35.309), *p* < 0.0001] was found to be an independent predictive factor for conservative treatment failure. The results of univariate and multivariate analyses of predictors for overall ostomy formation are demonstrated in Table 4 and revealed that BMI, ASA score, arterial hypertension, chronic kidney disease, immunosuppression, abscess size, failed medical therapy/urgent-emergency surgery, and conversion to open surgery were associated with overall ostomy formation (*p* < 0.05). In the multivariate analysis, BMI [OR 1.366, 95% CI (1.038–1.797), *p* = 0.026] remained the only significant influencing variable on overall ostomy formation in patients with diverticular abscess.

**Table 3:** Uni-and multivariate analyses of predictive risk factors of medical therapy failure/urgent-emergency surgery

	Failed medical therapy/urgent-emergency surgery (*n* = 18)	No failed medical therapy/urgent-emergency surgery (*n* = 87)	*p* value	Odds ratio (95% CI)	*p* value
**Variables**	Univariate analysis			Multivariate analysis	
Gender (*n*; %)			1.00		
Male Female	10 (55.56) 8 (44.44)	46 (52.87) 41 (47.13)			
Age (years) (mean ± SD)	51.555 ± 13.156	56.356 ± 11.928	0.130		
BMI (kg/m^2^) (mean ± SD)	29.156 ± 8.269	27.431 ± 6.183	0.314		
ASA (*n*; %)			0.186		
I II III IV	1 (5.56) 9 (50.0) 8 (44.44) 0 (0)	20 (22.99) 44 (50.57) 21 (24.14) 2 (2.30)			
Diabetes mellitus (*n*; %)			0.344		
Yes No	2 (11.11) 16 (88.89)	5 (5.75) 82 (94.25)			
Arterial hypertension (*n*; %)			0.010	0.407 (0.115–1.441)	0.163
Yes No	13 (72.22) 5 (27.78)	33 (37.93) 54 (62.07)			
Chronic kidney disease (*n*; %)			0.007	0.257 (0.048–1.393)	0.115
Yes No	5 (27.78) 13 (72.22)	4 (4.60) 83 (95.40)			
Immunosuppression (*n*; %)			0.130		
Yes No	5 (27.78) 13 (72.22)	10 (11.49) 77 (88.51)			
CRP (mg/dl) (mean ± SD)	8.516 ± 8.385	5.927 ± 7.655	0.202		
WBC (× 1000/µl) (mean ± SD)	12.638 ± 5.801	10.300 ± 4.611	0.065		
Hemoglobin (g/dl) (mean ± SD)	12.677 ± 2.236	13.460 ± 2.053	0.151		
Thrombocytes (× 1000/µl) (mean ± SD)	328.277 ± 111.098	291.494 ± 150.769	0.330		
Number of flairs (mean ± SD)	1.722 ± 0.894	1.724 ± 0.960	0.994		
Abscess size (*n*; %)			< 0.0001	**9.904 (2.778**–**35.309)**	** < 0.0001**
Micro Macro	4 (22.22) 14 (77.78)	69 (79.31) 18 (20.69)			

*ASA* American Society of Anesthesiologists, *BMI* body mass index, *CRP* C-reactive protein, *WBC* white blood count

**Table 4:** Uni-and multivariate analyses of predictive risk factors for overall ostomy formation

	Overall ostomy formation (*n* = 22)	No ostomy formation (*n* = 83)	*p* value	Odds ratio (95% CI)	*p* value
**Variables**	Univariate analysis			Multivariate analysis	
Gender (*n*; %)			0.233		
Male Female	9 (40.91) 13 (59.09)	47 (56.63) 36 (43.37)			
Age (years) (mean ± SD)	57.272 ± 10.985	55.072 ± 12.544	0.455		
BMI (kg/m^2^) (mean ± SD)	30.753 ± 7.514	26.918 ± 6.102	0.014	**1.366 (1.038**–**1.797)**	**0.026**
ASA (*n*; %)			0.035	3.561 (0.677–18.728)	0.134
I II III IV	1 (4.55) 10 (45.45) 10 (45.45) 1 (4.55)	20 (24.10) 43 (51.81) 19 (22.89) 1 (1.20)			
Diabetes mellitus (*n*; %)			0.635		
Yes No	2 (9.09) 20 (90.91)	5 (6.02) 78 (93.98)			
Arterial hypertension (*n*; %)			0.001	1.393 (0.155–12.532)	0.767
Yes No	17 (77.27) 5 (22.73)	29 (34.94) 54 (65.06)			
Chronic kidney disease (*n*; %)			0.019	30.977 (0–inf)	0.999
Yes No	5 (22.73) 17 (77.27)	4 (4.82) 79 (95.18)			
Immunosuppression (*n*; %)			0.015	19.349 (0–inf)	0.999
Yes No	7 (31.82) 15 (68.18)	8 (9.64) 75 (90.36)			
CRP (mg/dl) (mean ± SD)	8.781 ± 8.932	5.730 ± 7.403	0.104		
WBC (× 1000/µl) (mean ± SD)	12.295 ± 4.169	10.278 ± 5.001	0.086		
Hemoglobin (g/dl) (mean ± SD)	12.845 ± 2.111	13.453 ± 2.086	0.230		
Thrombocytes (× 1000/µl) (mean ± SD)	293.318 ± 86.524	299.172 ± 157.383	0.867		
Number of flairs (mean ± SD)	1.590 ± 0.734	1.759 ± 0.994	0.461		
Abscess size (*n*; %)			0.002	6.789 (0.601–76.646)	0.121
Micro Macro	9 (40.91) 13 (59.09)	64 (77.11) 19 (22.89)			
Failed conservative therapy/urgent-emergency surgery (*n*; %)			< 0.0001	8.003 (0.527–121.458)	0.134
Yes No	10 (45.45) 12 (54.55)	8 (9.64) 75 (90.36)			
Time interval surgery to last attack (days) (mean ± SD)	15.454 ± 22.079	25.253 ± 24.375	0.091		
Operative time (min) (mean ± SD)	284.954 ± 79.012	279.036 ± 79.270	0.756		
Conversion to open surgery (*n*; %)			0.027	4.716 (0.455–48.863)	0.194
Yes No	5/9 (55.56) 4/9 (44.44)	12/64 (18.75) 52/64 (81.25)			

*ASA* American Society of Anesthesiologists, *BMI* body mass index, *CRP* C-reactive protein, *WBC* white blood count

## Discussion

Our analysis including 105 patients with diverticular abscess undergoing primary medical treatment followed by surgical resection clearly demonstrated that an abscess larger than 3 cm in diameter (macro-abscess) is the only significant predictive factor of treatment failure defined as recurrent or persistent abscess, clinical deterioration, and subsequent urgent or emergency surgery. Furthermore, macro-abscess presence was significantly associated with higher conversion rates to open surgery, higher overall ostomy formation, and a prolonged total and postoperative hospital stay when compared to smaller abscesses (≤ 3 cm). Noteworthily, in the macro-abscess group, the choice of either percutaneous drainage or antibiotic alone did not affect the perioperative outcome in terms of conservative treatment failure or postoperative morbidities while a prolonged total hospital stay was associated with abscess drainage insertion. Multivariate analyses revealed that overall ostomy formation was significantly correlated with an increased BMI. Among the most commonly used sigmoid diverticulitis classifications (Table 2 suppl.), abscess diameter is explicitly mentioned in the World Society of Emergency Surgery (WSES) and Classification of Diverticular Disease (CDD) grading systems, and in the studies of Sallinen et al. and Mora Lopez et al. as a parameter for the severity of inflammation [1, 12, 13, 20–27]. The proposed diameter values for diverticular abscess stratification are mainly based on prognostic factors such as recurrence and treatment failure rates and technical feasibility of drainage insertion. However, the defined cut-off size varies between current guidelines as different scientific sources were considered with conflicting results and evidence levels ranging from moderate to low [11]. For example, the German [12, 13], ASCRS [2], and NICE [28] guidelines recommend drainage of abscess collections larger than 3 cm whereas smaller abscesses could be sufficiently managed with antibiotics alone not exposing patients at risk of emergency surgery or recurrent disease. In contrast, the EAES/SAGES [29] and WSES [30] societies stated that primary abscess sizes > 4 cm should be evaluated for drainage placement. Our work represents the second study of its type to analyze perioperative outcomes in relation to an abscess cut-off size of 3 cm, initially managed conservatively. In the study by Elagali et al. [14], 32 patients were treated with antibiotics and 114 patients with drainage for abscesses ≥ 3 cm prior surgery. No significant difference in treatment failure and subsequent urgent resection rates (*p* = 0.21) was found between both groups which is in line with our observation in the macro-abscess group and the results of a large meta-analysis [4]. However, we could not observe significantly higher postoperative complications in the antibiotics group as stated by Elagali et al. [14]. These findings raise the question whether percutaneous abscess drainage is always necessary or could be omitted in larger abscesses especially in the view of limited interventional radiology capacities and the potential complications associated with drainage insertion which range between 0 and 15% [3, 16]. Indeed, Siewert al. [31] showed that patients with abscess sizes between 3 and 4 cm can be effectively treated with antibiotics alone and Mali et al. [16] confirmed this observation even in abscess sizes ≥ 4 cm. Another study recommended a limit of two attempts for drainage placement in persistent diverticular abscess to avoid potential morbidity and ostomy creation with increased healthcare costs [32]. Based on our analysis, an increased BMI at the time of surgery for diverticular abscess requires a higher rate of ostomy formation, an observation which is further supported by an American study including 114 patients with abscess drainage [33]. Another large American database query [34] also revealed obesity as an independent risk factor of ostomy creation in diverticulitis. The purpose of ostomy formation is to avoid potential serious complications in obese high-risk patients. One important issue that must be addressed is the heterogeneity in definition of medical treatment failure across the available studies with reported abscess-related adverse events ranging from 30 days to 6 months after index admission [35–37]. A recently published meta-analysis [6] defined failure of non-operative management as persistent/aggravated abscess and/or sepsis, occurrence of abscess-caused complications, and the necessity of urgent or emergency surgery within 30–90 days from index admission. The pooled rate of treatment failure was 16.4% (12.6–20.2%). Our definition of conservative management failure is similar except the shorter fixed time interval of 30 days since admission. The overall failure rate in our cohort was 17.14%, which could admittedly represent an underreporting since the majority of the elective cases (67.81%) were operated within 30 days after the last flair. In the current guidelines, elective colectomy should be considered after successful medical treatment of acute complicated diverticulitis, particularly with regard to concomitant risk constellations [2, 12, 13]. Drainage insertion is significantly associated with an increased risk of recurrence compared to antibiotic therapy (23.6% versus 15.5%, *p* = 0.0001) in a large Danish register-based cohort study with long-term follow-up data [38] while an abscess size ≥ 5 cm is a risk factor for emergency surgery on short-term basis [8]. Although conservative therapy is an approved and feasible option after initial successful diverticular abscess treatment in selected cases [35, 39, 40], patients with previous diverticular abscess are more likely to present with a complicated recurrent flair at a substantial rate [9, 17]. The long-term mortality risk of recurrent diverticulitis with non-operative therapy is 2.0% compared with only 0.6% after surgery [35, 38]. On the other side, high mortality rates (4.6–7.21%) are observed after emergency surgery for initial medical treatment failure and recurrent attacks during second admission [5, 17]. In contrast, our study found a 0% mortality rate after elective and emergency surgery.

There are weaknesses inherent in the presented study. The retrospective design with a small study cohort qualifies for a downgraded evidence level. Lack of randomization and non-standardized therapy allocation mainly influenced by personal preference represent potential selection bias. Based on the eligibility criteria and our institutional approach in favor of interval sigmoidectomy, we were unable to draw comparative conclusions between successful non-surgical management followed by observation versus definitive surgical therapy on long-term follow-up analysis. Furthermore, there is still no consensus in the literature regarding the most suitable medical therapy (percutaneous drainage or antibiotics alone) in the management of larger diverticular abscess formations [4]. These questions are most accurately answered in the setting of large multicenter studies with unified treatment protocols and long-term follow-up data. The current German sigmoid diverticulitis classification system (CDD) appears to adequately stratify patients presenting with diverticular abscess formation according to the defined abscess size cut-off values.

## Conclusion

An abscess diameter larger than 3 cm is a predictive risk factor of non-operative treatment failure. Both antibiotic therapy and percutaneous drainage are feasible and safe options in the management of larger abscesses with similar perioperative outcomes and postoperative complications. In patients undergoing surgery for diverticular abscess, the risk of overall stoma formation increases with a higher BMI. Larger multicenter studies could provide valuable answers to the remaining uncertainties in the treatment of acute diverticulitis complicated by abscess.

## Supplementary Information

Below is the link to the electronic supplementary material.

Supplementary file1 (DOCX 31 KB)

Supplementary file2 (DOCX 13 KB)

Supplementary file3 (DOCX 21.8 KB)

## Data Availability

The datasets used and/or analyzed during the current study are available from the corresponding authors on reasonable request.
