# Supplementary material for: Clinical relevance of the 3-cm threshold in sigmoid diverticulitis with abscess: consensus or quandary?
Source: Int J Colorectal Dis. 2024 Jul 12;39(1):106. doi: 10.1007/s00384-024-04682-z (PMC11245413; doi:10.1007/s00384-024-04682-z)
Supplement: Supplementary file 2 — Supplementary file2 (DOCX 13 KB) [file 384_2024_4682_MOESM2_ESM.docx]

| Table 1 suppl. Perioperative course and operative morbidity of drainage versus antibiotics in group B | | | |
| --- | --- | --- | --- |
|  | **Drainage**  **n= 18** | **Antibiotics**  **n= 14** | **P-Value** |
| **Variables** |  |  |  |
| Failed conservative therapy/urgent-emergency surgery, (n; %) | 10 (55.56) | 4 (28.57) | 0.165 |
| Abscess recurrence (n; %) | 4 (22.22) | 0 (0) | 0.113 |
| Route of access, (n; %)  Open  Laparoscopic | 8 (44.44)  10 (55.56) | 8 (57.14)  6 (42.86) | 0.722 |
| Conversion to open surgery, (n; %) | 6/10 (60.0) | 1/6 (16.67) | 0.145 |
| Secondary ostomy, (n; %) | 1 (5.56) | 0 (0) | 1.000 |
| Overall ostomy formation, (n; %) | 8 (44.44) | 5 (35.71) | 0.725 |
| Overall postop. morbidity, (n; %) | 10 (55.56) | 7 (50.0) | 1.000 |
| Major morbidity (CD ≥ 3a), (n; %) | 6 (33.33) | 3 (21.43) | 0.694 |
| Wound infection, (n; %) | 8 (44.44) | 7 (50.0) | 1.000 |
| Anastomotic leak, (n; %) | 1/14 (7.14) | 0/12 (0) | 1.000 |
| Re-operation, (n; %) | 2 (11.11) | 3 (21.43) | 0.631 |
| Postoperative ileus, (n; %) | 3 (16.67) | 1 (7.14) | 0.613 |
| Intra-abdominal abscess, (n; %) | 2 (11.11) | 0 (0) | 0.492 |
| Trocar/incisional hernia, (n; %) | 1 (5.56) | 0 (0) | 1.000 |
| Ureter lesion, (n; %) | 0 (0) | 1 (7.14) | 0.437 |
| Postop. LOS (days), (mean ± SD) | 22.166 ± 21.038 | 13.142 ± 6.937 | 0.103 |
| Total LOS (days), (mean ± SD) | 32.777 ± 21.810 | 20.785 ± 8.911 | **0.045** |
| CD: Clavien-Dindo, LOS: length of hospital stay | | | |
