# Supplementary material for: Clinical relevance of the 3-cm threshold in sigmoid diverticulitis with abscess: consensus or quandary?
Source: Int J Colorectal Dis. 2024 Jul 12;39(1):106. doi: 10.1007/s00384-024-04682-z (PMC11245413; doi:10.1007/s00384-024-04682-z)
Supplement: Supplementary file 3 — Supplementary file3 (DOCX 21.8 KB) [file 384_2024_4682_MOESM3_ESM.docx]

| Table 2 suppl. Various sigmoid diverticulitis grading systems | |
| --- | --- |
| **Classification** | **Abscess size cut-off value (cm)** |
| Hinchey [20] | Not defined |
| Modified Hinchey by Wasvary et al. [22] | Not defined |
| Neff et al. [21] | Not defined |
| Ambrosetti et al. [23] | Not defined |
| Kaiser et al. [1] | Not defined |
| Modified Neff classification by Mora Lopez et al. [24] | Complicated diverticulitis 1b: < 4cm 2: > 4cm (pelvic abscess) |
| (WSES) Sartelli et al. [25] | Complicated diverticulitis 1B: ≤ 4cm 2A: > 4cm |
| Sallinen et al. [27] | Complicated diverticulitis 2: < 6cm (small abscess) 3: ≥ 6cm (large abscess) |
| Hansen/Stock [26] | Not defined |
| Classification of Diverticular Disease (CDD) [12, 13] | Complicated diverticulitis 2a: ≤ 3cm (micro-abscess) 2b: > 3cm (macro-abscess) |
